# The Macroecology of Chemical Communication in Lizards: Do Climatic Factors Drive the Evolution of Signalling Glands?

**DOI:** 10.1007/s11692-018-9447-x

**Published:** 2018-03-10

**Authors:** Manuel Jara, Alba Frias-De-Diego, Roberto García-Roa, Mónica Saldarriaga-Córdoba, Lilly P. Harvey, Rachel P. Hickcox, Daniel Pincheira-Donoso

**Affiliations:** 1 Laboratory of Evolutionary Ecology of Adaptations, School of Life Sciences, University of Lincoln, Brayford Campus, Lincoln, LN6 7DL UK; 2 Ethology Lab, Cavanilles Institute of Biodiversity and Evolutionary Biology, University of Valencia, Valencia, Spain; 3 Centro de Investigación en Recursos Naturales y Sustentabilidad, Universidad Bernardo O’Higgins, Santiago, Chile; 4 School of Science and Technology, Nottingham Trent University, Clifton Campus, Nottingham, NG11 8NS UK; 5 Present Address: Department of Fish and Wildlife Conservation, Virginia Tech, Blacksburg, VA USA

**Keywords:** Chemical communication, Signalling glands, Precloacal glands, Sexual selection, Macroecology, Lizards, *Liolaemus*

## Abstract

Chemical communication plays a pivotal role in shaping sexual and ecological interactions among animals. In lizards, fundamental mechanisms of sexual selection such as female mate choice have rarely been shown to be influenced by quantitative phenotypic traits (e.g., ornaments), while chemical signals have been found to potentially influence multiple forms of sexual and social interactions, including mate choice and territoriality. Chemical signals in lizards are secreted by glands primarily located on the edge of the cloacae (precloacal glands, PG) and thighs (femoral glands), and whose interspecific and interclade number ranges from 0 to > 100. However, elucidating the factors underlying the evolution of such remarkable variation remains an elusive endeavour. Competing hypotheses suggest a dominant role for phylogenetic conservatism (i.e., species within clades share similar numbers of glands) or for natural selection (i.e., their adaptive diversification results in deviating numbers of glands from ancestors). Using the prolific *Liolaemus* lizard radiation from South America (where PG vary from 0 to 14), we present one of the largest-scale tests of both hypotheses to date. Based on climatic and phylogenetic modelling, we show a clear role for both phylogenetic inertia and adaptation underlying gland variation: (i) solar radiation, net primary productivity, topographic heterogeneity and precipitation range have a significant effect on PG variation, (ii) humid and cold environments tend to concentrate species with a higher number of glands, (iii) there is a strong phylogenetic signal that tends to conserve the number of PG within clades. Collectively, our study confirms that the inertia of niche conservatism can be broken down by the need of species facing different selection regimes to adjust their glands to suit the demands of their specific environments.

**Electronic supplementary material:**

The online version of this article (10.1007/s11692-018-9447-x) contains supplementary material, which is available to authorized users.

## Introduction

Communication underlies most ecological and social interactions among living organisms, and therefore, its evolution influences most life history, developmental, sensory and cognitive processes (Andersson 1994; Roff 2002; Searcy and Nowicki 2005; Westneat and Fox 2010). Although studies of animal communication have primarily focused on the role that visual (trait displays) and acoustic (sounds in specific frequencies and patterns) signals play in shaping interactions, the role of chemical communication has gained an increasingly more central role in understanding the evolution of social and sexual dynamics within populations (Alberts 1992; Schwenk 1995; Martin and Lopez 2000; Kratochvil and Frynta 2002; Ibáñez et al. 2017; MacGregor et al. 2017). The evolution of traits responsible for chemical communication has been suggested to be driven by selection arising from competition over mates (sexual selection), and from ecological pressures that affect signal efficiency (Alberts 1992; Escobar et al. 2001; Baeckens et al. 2017b; García-Roa et al. 2017c). As a result, the hypothesis that variation in the number of signalling glands is shaped by geographic gradients of climatic factors that affect the efficiency of the signal delivery, thus resulting in macroecological patterns of variation in chemical phenotypes, has been suggested (Escobar et al. 2003). However, this hypothesis has only rarely been tested, and the limited available evidence is highly conflicting (Pincheira-Donoso et al. 2008a; Baeckens et al. 2015).

In lizards, several chemical signals for communication are produced by different systems of epidermic glands, primarily found on the posterior edge of the cloacae (precloacal glands, PG hereafter) and on the ventral surface of the thighs (femoral glands) (Cole 1966; Pincheira-Donoso et al. 2008a; García-Roa et al. 2017a). Accumulating evidence has reinforced the idea that the chemical signals secreted by these glands play a pivotal role in lizard sexual communication (but see MacGregor et al. 2017; García-Roa and Carazo 2017). For example, while female mate choice based on quantitative phenotypic traits has been difficult to demonstrate in these organisms (Olsson and Madsen 1995; Olsson et al. 1998), a number of studies have suggested that female mating decisions are influenced by chemical signals produced by males (Schwenk 1995; Martin and Lopez 2000; López et al. 2002, 2003). In fact, the highly debated theory that sexual selection drives speciation (Panhuis et al. 2001; Ritchie 2007) has more recently invoked the influence of scents as a potential component of the factors involved in the formation of new species (Martin and Lopez 2006; Labra 2011; Pincheira-Donoso 2012; Baeckens et al. 2017a). As a result, there has been emerging interest in investigating the effects that sexual and ecological pressures exert on the evolution of signalling glands.

Only a few studies have explored the role that ecological pressures play in the evolution of signalling glands. The first comprehensive comparative analysis investigating the relationship between climatic factors and the number of PG, conducted in the South American *Liolaemus* radiation, concluded that the number of glands increases towards warmer and windier climates (Escobar et al. 2001). However, this study entirely lacked phylogenetic control. In an attempt to assess the implications of this analytical limitation, a subsequent study employing phylogenetic analyses revealed that a strong phylogenetic signal explains variation in gland numbers among *Liolaemus* species, while no signals of environmental (i.e., adaptive) effects were identified (Pincheira-Donoso et al. 2008a). More recently, a comparative study on lacertid lizard species showed that numbers of femoral glands do not respond to climatic gradients. Instead, a significant relationship was found between use of microhabitats and variation in the number of these glands (Baeckens et al. 2015). As a result, there has been an emerging interest in identifying the extent to which shared ancestry and climatic factors operating on signal delivery interact to shape the evolution of signalling glands (Escobar et al. 2001; Pincheira-Donoso et al. 2008a; Iraeta et al. 2011; Baeckens et al. 2015; Mayerl et al. 2015; García-Roa et al. 2017a). However, only a handful of empirical studies that combine both, environmental factors and phylogeny, are currently known.

In this study, we suggest that both phylogenetic and climatic factors are likely to play a role in determining the number of glands needed for effective chemical signalling. The need for testing this hypothesis stems from the fact that although numbers of PG differ between clades, they also show variation among species within the same clades (Pincheira-Donoso et al. 2008a; García-Roa et al. 2017a). We address the macroecological hypothesis that the evolution of signalling glands is driven by selection arising from climatic factors varying through geographic space. We use the *Liolaemus* radiation as a model system, in which only PG exist. Within this genus, the number of PG varies from 0 to 14, with multiple cases of phylogenetically independent episodes of PG losses (Videla and Cei 1998; Pincheira-Donoso and Núñez 2005; Pincheira-Donoso and Scolaro 2007; Pincheira-Donoso et al. 2008a). In addition, *Liolaemus* embodies the second most prolific adaptive radiation of amniotes on Earth (Pincheira-Donoso et al. 2013a). This lineage has diversified into 270+ species adapted to the widest range of environmental and climatic conditions recorded for a single reptile genus (Pincheira-Donoso et al. 2008c, 2013b), ranging from the Atacama Desert to the Tierra del Fuego (the southernmost site where reptiles have been found), and from sea level to 5000+ m elevation (Espinoza et al. 2004; Pincheira-Donoso et al. 2008b; Labra et al. 2009; Pincheira-Donoso 2011). Across these extreme environments a great degree of phenotypic and ecological variation has evolved both among and within species (Schulte et al. 2004; Pincheira-Donoso et al. 2009, 2011), which makes this lineage an ideal model to test questions involving selection gradients affecting trait evolution in a macroecological perspective.

## Materials and Methods

### Species Distribution and Phylogenetic Data

**Fig. 1:**
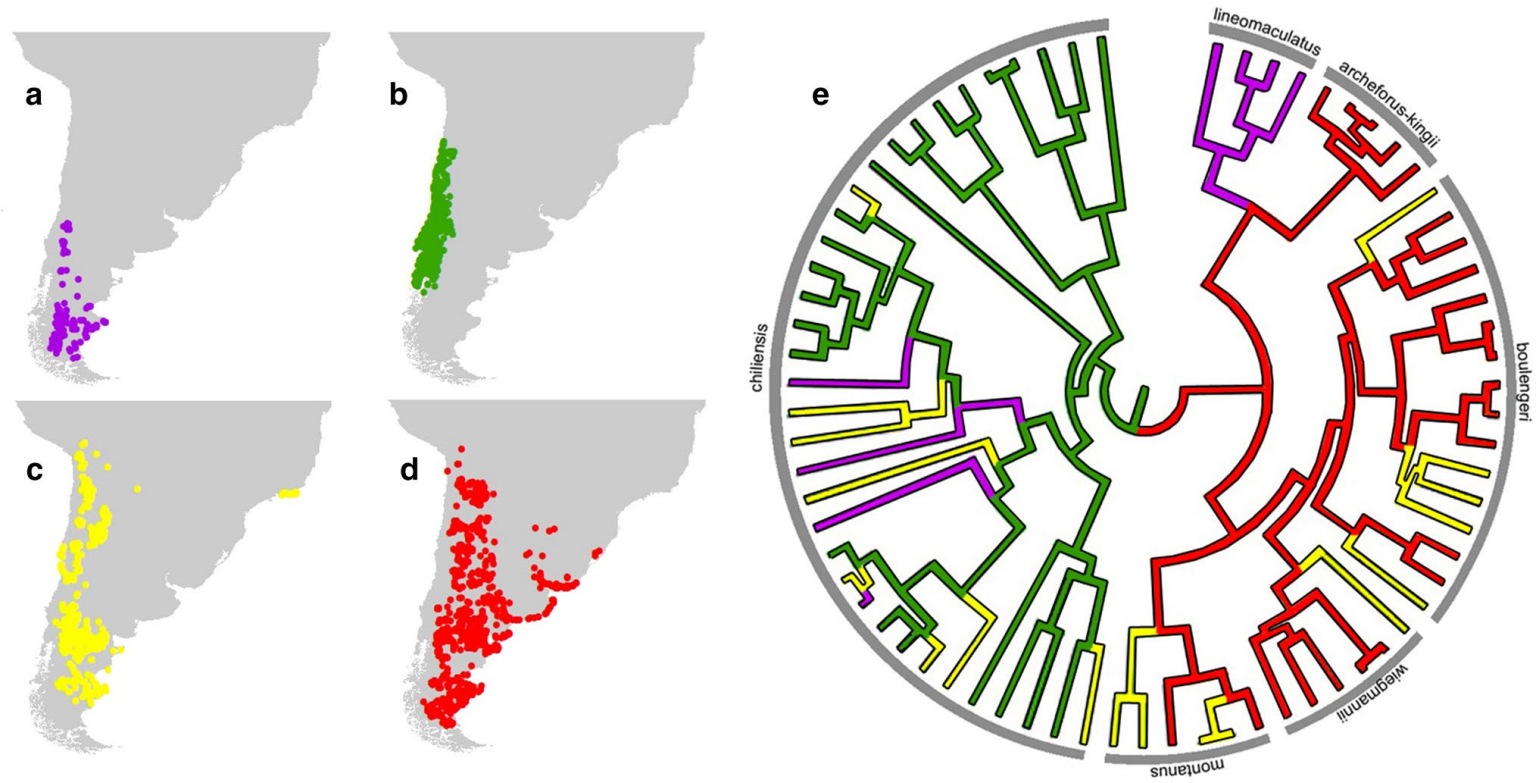
Distribution of precloacal gland (PG) numbers across species of the *Liolaemus* genus. **a** Species lacking PG, **b** species with 1–3 PG, **c** species with > 3–6 PG, and **d** species with > 6 PG. The phylogenetic tree (**e**) represents the distribution of PG numbers among *Liolaemus* in the phylogenetic space. Grey ring around phylogenetic tree represents the different *Liolaemus* clades

To investigate whether variation in PG number across species is driven by selection emerging from environmental factors, we modelled the multivariate climatic niche of 97 *Liolaemus* species, by extracting the average climatic conditions of all Global Positioning System (GPS) data available for each species (Supplementary Table S1). These data consist of 6612 GPS records obtained from 15 + years of fieldwork, museum samples and the literature (Cei 1986, 1993; Pincheira-Donoso and Núñez 2005; Scolaro 2005, 2006; Pincheira-Donoso et al. 2017). Data were analysed via multiple regression analyses using conventional and phylogenetic models. To correct for phylogenetic non-independence, we employed the most comprehensive multi-gene phylogenetic tree currently available for the Liolaemidae family (Pincheira-Donoso et al. 2017), which covers 72 species in our dataset (Fig. 1, Supplementary Table S1).

### Environmental Predictors

We created a dataset spanning a range of candidate environmental predictors (all Bio 19 variables, plus elevation; see below and Appendix). All these variables characterise climates during the period 1970–2000, and were obtained from the WorldClim2 data archive (Fick and Hijmans 2017), with a spatial resolution of 2.5 arc minutes (~ 5 km at the equator). We expanded the range of predictors in our dataset by adding levels of ultraviolet-B radiation (UV-B) and Net Primary Production (NPP; the net amount of solar energy converted to plant organic matter through photosynthesis—measured in units of elemental carbon per year, gC/m^2^/year), both of them extracted from NASA’s Earth Observing System database (available at: https://neo.sci.gsfc.nasa.gov ). Both UV-B and NPP were downloaded at the same spatial resolution as the WorldClim2 variables above.

In addition, we created two additional variables expected to affect the climatic regimes of species: climatic and topographic heterogeneity. To generate these variables we employed the species distribution modelling (SDM) toolbox v1.1b in ArcGIS v10.4 (Brown 2014). This tool performs a principal component analysis (PCA) from the input set of raster bands (i.e., the 19 climatic variables from WorldClim2), and generates a single multiband raster which depicts the climatic variation (i.e., heterogeneity) within a specified area (Brown 2014). While topographic heterogeneity represents the heterogeneity of the landscape, calculated by the elevation from each raster pixel and the eight cells neighbouring the focal cell (Brown 2014).

To reduce levels of collinearity among the environmental variables, we used variance inflation factors (VIF) implemented in the “usdm” R-package. Using this approach, we excluded all the highly-correlated variables from the model (VIF > 10) (Markwick et al. 2000). This method is based on the square of the multiple correlation coefficient (*R*^2^) resulting from regressing the predictor variable against all other predictor variables. Following this approach, we reduced the original dataset to the variables latitude, mean annual temperature (Bio 1), mean diurnal range (Bio 2; mean of monthly [maximum temperature–minimum temperature]), temperature annual range (Bio 7; max temperature of warmest month*–*min temperature of coldest month), mean annual precipitation (Bio 12), precipitation of wettest month (Bio 13), precipitation of driest month (Bio 14), precipitation annual range (precipitation of wettest month*–*precipitation of driest month), elevation, UV-B radiation, climatic heterogeneity, topographic heterogeneity, and NPP. To extract the environmental variables per each location, we used Spatial Analysis toolbox in ArcGIS v10.4.

### Spatial and Phylogenetic Analysis

We investigated the role that environmental drivers play in shaping variation in *Liolaemus* PG by implementing both spatial [ordinary least square models (OLS) using ArcGIS v10.4—these analyses included all 97 species in our dataset] and phylogenetic [phylogenetic generalized least square models (PGLS)—these analyses included the 72 species in the phylogeny] multiple regressions (Martins and Hansen 1997) using the R package ‘caper’ (Orme et al. 2012). Before we performed explicit tests of our core hypotheses, we addressed the question whether the number of PG could be an allometric function of variation in body size (e.g., higher numbers of PG are found in larger species with more body surface available to accommodate more glands). To quantify the extent to which body size influences gland numbers, we employed snout-vent length (SVL) as our proxy for size (the traditional measure of body size in lizards) and as the predictor of PG number in conventional OLS and in PGLS regressions.

We then performed our multiple regressions to determine the influence that environmental factors exert on the variation of PG numbers among *Liolaemus* species. As described above, we first tested the adaptive hypothesis using the entire dataset, without phylogenetic control via OLS spatial regression models that included the above set of selected environmental predictors (Wiens et al. 2006). The same analyses were then performed based on our phylogenetic tree. Finally, to estimate the magnitude of phylogenetic signal on PG, we employed two alternative metrics, Blomberg’s K (Blomberg et al. 2003) and Pagel’s λ (Pagel 1999), by performing analyses implemented in the R package “phytools” (Revell 2012), respectively. *K* returns values that express from zero (no phylogenetic signal in the trait) to 1, which indicates that there is strong phylogenetic signal and the trait has evolved according to the Brownian Motion model (Pincheira-Donoso et al. 2015), whereas *K* > 1 indicates that close relatives evolve similar traits than expected under a Brownian Motion model. On the other hand, Pagel’s λ varies from 0 to 1, where λ = 0 indicates no phylogenetic signal, and λ = 1 indicates strong phylogenetic signal, and thus, that the trait has evolved according to the Brownian motion model (Kamilar and Cooper 2013).

## Results

Our results reveal that *Liolaemus* species show a strong pattern of variation in PG numbers across geographic regions. We found that species lacking PG are mostly located in cold environments (Andean-Patagonian ecoregions, Fig. 1a), while species with a low number of PG (1–3 glands) are distributed on the west side of the Andes, in Chile (Fig. 1b). In contrast, the east side of the Andes, in Argentina, concentrates most species with higher PG numbers, above > 3 glands (Fig. 1c, d).

### Test of Phylogenetic Signal

We observed a significant phylogenetic signal when estimated using both Blomberg’s *K* (*K* = 1.17, *P* = 0.001) and Pagel’s λ (λ = 0.98, *P* < 0.001). Some clear clusters of high and low values of PG number associated with the different clades of *Liolaemus* were observed (Fig. 1b). The lowest numbers of PG were found in the *chiliensis* and *lineomaculatus* clades (in this last lineage all species lack PG, and thus, it is observed to be a derived condition resulting from a secondary loss of glands). In contrast, the highest values were mostly observed in the of the *montanus* and *boulengeri* clades (Fig. 1).

### Spatial Analysis

The effects of body size on the variation of PG numbers, considering spatial autocorrelation, were very low among *Liolaemus* lizards (OLS, *R*^2^ = − 0.01, *P* < 0.001). Here we observed that: precipitation range (OLS, *R*^2^ = 0.16, *P* < 0.001), topographic heterogeneity (OLS, *R*^2^ = 0.14, *P* < 0.001), precipitation of wettest month (OLS, *R*^*2*^ = 0.14, *P* < 0.001) and mean annual precipitation (OLS, *R*^2^ = 0.11, *P* < 0.001) showed a negative relationship with the increasing number of SG. Thus, these *R*^2^ values increased with the combination of the model, ranging from 23% for pairs to a 36% for the best combination of four variables (solar radiation, topographic heterogeneity, net primary productivity and precipitation range), where solar radiation and NPP showed a positive relationship with PG number (Table 1).

**Table 1:** Spatial relationship (OLS regression) among precloacal gland (PG) numbers (as response variable in all the analyses) in *Liolaemus* lizards and different environmental predictor variables

Predictor 1	Predictor 2	Predictor 3	Predictor 4	*R* ^2^	AICc	VIF	*P*
+Latitude	–	–	–	0.03	452.65	–	*0.000*
+Bio 1	–	–	–	0.02	452.77	–	*0.000*
+Bio 2	–	–	–	0.03	452.03	–	*0.000*
+Bio 7	–	–	–	0.04	451.29	–	*0.000*
−Bio 12	–	–	–	0.11	444.48	–	*0.000*
−Bio 13	–	–	–	0.14	440.52	–	*0.000*
−Bio 14	–	–	–	0.00	454.95	–	*0.000*
−Prec_range	–	–	–	0.16	438.54	–	*0.000*
+Elevation	–	–	–	0.00	455.33	–	*0.000*
+Clim_het	–	–	–	0.00	455.49	–	*0.000*
−Topo_het	–	–	–	0.14	440.98	–	*0.000*
+NPP	–	–	–	0.01	456.10	–	*0.000*
+UV-B radiation	–	–	–	0.05	450.13	–	*0.000*
+NPP	−Prec_range	–	–	0.23	431.36	1.34	*0.000*
+Bio 7	−Prec_range	–	–	0.23	431.65	1.01	*0.000*
−Bio 13	+NPP	–	–	0.23	431.90	1.45	*0.000*
+Bio 2	+NPP	−Prec_range	–	0.32	421.20	1.42	*0.000*
+UV-B radiation	+NPP	−Prec_range	–	0.31	422.49	1.77	*0.000*
+Bio 2	−Bio 13	+NPP	–	0.29	424.65	1.51	*0.000*
+UV-B radiation	−Topo_het	+NPP	−Prec_range	0.36	416.26	1.84	*0.000*
−Bio 13	+UV-B radiation	−Topo_het	+NPP	0.35	417.06	1.90	*0.000*
+Bio 2	−Topo_het	+NPP	−Prec_range	0.35	417.54	1.69	*0.000*

Italic indicates significant relationships are represented by *P* values

*Bio 1* mean annual temperature, *Bio 2* temperature diurnal range, *Bio 7* temperature annual range, *Bio 12* mean annual precipitation, *Bio 13* precipitation of wettest month, *Bio 14* precipitation of driest month, *Prec_range* precipitation annual range, *Clim_het* climatic heterogeneity, *Topo_het* topographic heterogeneity and *NPP* net primary productivity. *AICc* Akaike’s Information Criterion *VIF* max variance inflation factor, model variable sign appears before the name of the variable (±)

### Phylogenetic Regressions

We firstly tested the potential allometric effects of body size on PG numbers. These analyses failed to identify a role for body size on PG when tests were performed with phylogenetic control (PGLS, *R*^2^ = − 0.02, *P* < 0.65).

PGLS models containing the whole range of selected predictors revealed low individual contribution of the environmental factor variables (up to 4%), which, in contrast, increase significantly when combined (Table 2). The combination values varied from 11% for pairs to 40% for combinations of four environmental predictors (see Table 2) with the combination of UV-B radiation, topographic heterogeneity, NPP and precipitation range, as the best predictors of PG variation.

**Table 2:** Phylogenetic generalised least squares (PGLS) regression among precloacal gland (PG) numbers (as response variable in all the analyses) in *Liolaemus* lizards and different environmental predictor variables

Predictor 1	Predictor 2	Predictor 3	Predictor 4	*R* ^2^	*F*	*P*
+Latitude	–	–	–	0.01	0.44	0.52
+Bio 1	–	–	–	0.02	1.14	0.29
+Bio 2	–	–	–	0.02	1.25	0.27
+Bio 7	–	–	–	0.02	1.15	0.29
−Bio 12	–	–	–	0.03	2.21	0.14
−Bio 13	–	–	–	0.03	2.29	0.13
−Bio 14	–	–	–	0.01	0.59	0.45
−Prec_range	–	–	–	0.03	2.37	0.13
+Elevation	–	–	–	0.00	0.03	0.86
+Clim_het	–	–	–	0.01	0.46	0.50
−Topo_het	–	–	–	0.04	2.91	0.09
+NPP	–	–	–	0.00	0.14	0.71
+UV-B radiation	–	–	–	0.01	1.02	0.32
+Bio 2	−Bio 12	–	–	0.17	4.54	*0.01*
−Bio 12	+Elevation	–	–	0.14	3.67	*0.02*
+Bio 2	−Bio 13	–	–	0.11	2.90	*0.04*
+Bio 2	−Bio 12	+Elevation	–	0.27	3.35	*0.00*
+Bio 2	−Bio 13	+Elevation	–	0.25	3.04	*0.01*
+Bio 2	−Bio 12	−Bio 13	–	0.22	2.55	*0.02*
+UV-B radiation	−Topo_het	+NPP	−Prec_range	0.40	2.54	*0.01*
+Bio 2	+Bio 7	−Bio 12	+NPP	0.39	2.34	*0.01*
+Bio 2	−Bio 12	−Bio 13	+Elevation	0.38	2.29	*0.01*

Italic indicates significant relationships are represented by *P* values

*Bio 1* mean annual temperature, *Bio 2* temperature diurnal range, *Bio 7* temperature annual range, *Bio 12* mean annual precipitation, *Bio 13* precipitation of wettest month, *Bio 14* precipitation of driest month, *Prec_range* precipitation annual range, *Clim_het* climatic heterogeneity, *Topo_het* topographic heterogeneity and *NPP* net primary productivity. *AICc* Akaike’s Information Criterion *VIF* max variance inflation factor, model variable sign appears before the name of the variable (±)

## Discussion

Our study provides one of the few comparative analyses investigating the macroecological drivers behind the evolution of lizard signalling glands—a primary functional system employed by these reptiles to engage in social and sexual chemical communication. The prolific radiation of *Liolaemus* lizards has served as a primary model system for studies addressing hypotheses about the factors driving evolution of these glands. However, such studies have resulted in entirely opposite conclusions, mostly given the intrinsic limitations in their analytical resources. While the very first comparative (but not-phylogenetically controlled) study (Escobar et al. 2003) consolidated the hypothesis that PG are shaped by natural selection emerging from climatic factors operating on signal efficiency, a subsequent study (Pincheira-Donoso et al. 2008a), based on phylogenetic analyses, concluded that the main factor explaining variation in glands is phylogenetic inertia. Our study, based on a considerably larger number of species, on a comprehensive molecular phylogeny, and in robust GIS-based measures of multiple climatic factors, confirms the strong influence of phylogenetic inertia [see also (García-Roa et al. 2017a), for lizards globally], but also, identifies the role that some climatic factors (solar radiation, topographic heterogeneity, productivity and precipitation range) play in driving variation *within* subclades of *Liolaemus*. Therefore, the expanded analytical strength offered by our study provides a more inclusive conclusion that combines the contribution of shared ancestry in interactions with environmental factors as the elements shaping the extreme diversity observed in these glands in *Liolaemus* (Pincheira-Donoso et al. 2008a) and in lizards globally (García-Roa et al. 2017a).

### Teasing Apart the Adaptive and the Phylogenetic Signal

Our findings suggest a balanced scenario where phylogenetic relatedness strongly influences the overall range of PG across lineages, but where the accumulation of variation across species within clades [which exists and is important; see Supplementary Table 1 (Pincheira-Donoso et al. 2008a; García-Roa et al. 2017a)] is likely to result from species adaptations to local environmental factors that potentially affect the chemical stability of their scents. In the context of this hypothesis, the *Liolaemus* model offers unique advantages given their exceptional diversity in species richness, occupation of diverse climatic/geographic settings, species ecology, and PG numbers—these features offer substantial degrees of variation to address our core question about the role of environmental gradients in driving predictable PG variation. On the other hand, we suggest that a more in-depth mechanistic understanding of the above hypothesis, and thus of the contributions of phylogenetic conservatism and accumulation of adaptive variation in shaping signalling gland numbers, will require further tests in other groups of lizards (García-Roa et al. 2017b). In fact, despite the above advantages of *Liolaemus*, a limitation intrinsic to this model system is that its different major subclades tend to occupy different geographic/climatic regions (Harmon et al. 2003; Pincheira-Donoso et al. 2008a). Therefore, the amount of environmental variation ‘available’ within each clade is not as substantial as it is in other clades of lizards with more widespread geographic/climatic distributions. In line with this observation, the *chiliensis* clade is the lineage with the widest-spread distribution within *Liolaemus*, and this clade spans three of the four categories of PG variation shown in Fig. 1 (while the other clades span lower ranges of relative variance in PG). Other lizard clades where species span a wide range of environmental conditions will be exposed to greater degrees of variance in natural selection regimes across phylogenetically related species, which is predicted to promote greater degrees of PG disparity across species.

Additional insights on the role of natural selection in shaping signalling glands (femoral glands in this case) were recently revealed by a comparative study on lacertid lizards (Baeckens et al. 2015). Although Baeckens et al.’s analyses failed to identify evidence for climatic factors as predictors of femoral gland variation (under phylogenetically controlled tests), these authors showed that interspecific differences in microhabitat occupation partly predicts variance in the numbers of glands. Essentially, Baeckens et al. (2015) suggested that both transmission and quality of the chemical scents are expected to be altered by the physical conditions offered by different substrate structures. The same conclusion was previously drawn from studies conducted in other organisms, which showed that a functional affinity between the produced scents and the substrate in which it is deposited for subsequent transmission is crucial for signal delivery and fade-out times (Regnier and Goodwin 1977; Alberts 1992; Elias et al. 2004). Collectively, therefore, these studies show that there is a local-scale ecological component that affects gland variation, and that this ‘microecological’ signal can be obscured at macroecological scales.

Collectively, the accumulation of evidence raises the possibility that natural selection on signalling glands may emerge from the interaction between the physical properties of microhabitat structures where scents are spread on, and the climatic factors that influence the physical properties that microhabitat substrates exert on scents (Baeckens et al. 2017a). For example, the surface of the same type of microhabitat structure (e.g., the surface of a rock) is expected to offer different physical properties in a desert, where temperatures and solar radiation are high, and humidity is low, compared to a temperate forest, in which these climatic conditions are entirely the opposite. These different combinations of microhabitats and climate along geographic space are, therefore, likely to create different regimes of natural selection on chemical signals, leading to adaptive clines in the traits implicated with effective signal delivery (Escobar et al. 2003; Baeckens et al. 2015). As discussed above, we argue that the signal of divergent regimes of selection will strengthen as the range of climatic conditions (across areas) and of microhabitat structures (within the same area) covered by the species of a lineage increase. These hypotheses that have only recently started to emerge as more phylogenetic comparative studies appear, potentially offer promising starting points to elucidate the contributions that adaptation and phylogenetic conservatism play in the evolution of these traits that operate as social glue in lizards.

Finally, why the loss of PG in multiple independent evolutionary episodes has taken place in *Liolaemus* (Fig. 1), and in many other lizard lineages globally (García-Roa et al. 2017a), remains an open question that offers a potentially interesting perspective to tease apart the role of selection and shared ancestry in the evolution of this trait. The only visible pattern we observe is that species lacking glands are strongly restricted to Andean-Patagonian climates (Fig. 1), while species with glands occupy a range of highly contrasting environments. Given that the functionality of sexual traits depends on both a species mating system and their interaction with the environment mediated by their effects on fitness (Andersson 1994; Shuster and Wade 2003; Cornwallis and Uller 2010), we argue that climatic variables alone are unlikely to provide the answer. Instead, environmental factors in association with behavioural modes of sexual interaction may offer the multivariate context to explain gland loss. For example, male territoriality in geckos is strongly associated with head sexual dimorphism and with the presence of precloacal glands, while their secondary loss is associated with the lack of territoriality (Kratochvil and Frynta 2002). Therefore, a relationship between mating systems and the cold Andean-Patagonian climates may have driven the loss of glands in *Liolaemus*. However, data on mating systems within this lizard genus remain highly limited, and hence, a test of this hypothesis is impracticable at present. Yet, future studies on lizard groups in which mating systems are better studied (or large-scale studies using hundreds of species from multiple clades) may offer an excellent opportunity to identify the causes underlying the loss of a trait with paramount sexual and social importance.

### Electronic supplementary material

Below is the link to the electronic supplementary material.

Supplementary material 1 (DOCX 50 KB)

## Appendix

Climatic variables used in this study: Latitude, Bio 1 = Mean annual temperature, Bio 2 = Temperature diurnal range, Bio 7 = Temperature annual range (Max Temperature of Warmest Month–Min Temperature of Coldest Month), Bio 12 = Mean annual Precipitation, Bio 13 = Precipitation of wettest month, Bio 14 = Precipitation of driest month, Precipitation annual range (Precipitation of wettest month—Precipitation of driest month), Elevation, Climatic heterogeneity, Topographic heterogeneity, Net Primary Productivity (NPP), UV-B radiation.
