# Supplementary material for: The Macroecology of Chemical Communication in Lizards: Do Climatic Factors Drive the Evolution of Signalling Glands?
Source: Evol Biol. 2018 Mar 10;45(3):259–67. doi: 10.1007/s11692-018-9447-x (PMC6096677; doi:10.1007/s11692-018-9447-x)
Supplement: Supplementary file 1 — Supplementary material 1 (DOCX 50 KB) [file 11692_2018_9447_MOESM1_ESM.docx]

**SUPPLEMENTARY TABLE S1.**

**Supplementary Table S1**  Data used in this study, where SVL=Snout-Vent length (mm), PG = number of precloacal glands, Lat= Latitude, Bio 1= Mean annual temperature (°C), Bio 2= Temperature diurnal range (°C), Bio 7= Temperature annual range (°C), Bio 12= Mean annual precipitation (mm), Bio 13= Precipitation of wettest month (mm), Bio 14= Precipitation of driest month (mm), Prec_range= Precipitation annual range (mm), Clim_het= Climatic heterogeneity (%), Topo_het= Topographic heterogeneity (%), NPP= Net Primary Productivity ((gc/m²/yr) and UV-B= UV-B radiation (MJ m^−2^). Taxa for which phylogenetic information was available are indicated in bold.

| Species | SVL | PG | Lat | Bio1 | Bio2 | Bio7 | Bio12 | Bio 13 | Bio14 | Prec_range | Elevation | Clim_het | Topo_het | NPP | UV-B |
| --- | --- | --- | --- | --- | --- | --- | --- | --- | --- | --- | --- | --- | --- | --- | --- |
| ***L. abaucan*** | 60.7 | 5.5 | -27.5 | 15.5 | 16.1 | 28.7 | 105.0 | 28.0 | 1.0 | 27.0 | 1735.5 | 35.4 | 10.3 | 268.0 | 19934.1 |
| ***L. albiceps*** | 86.9 | 7.9 | -24.4 | 8.3 | 16.8 | 26.9 | 159.4 | 46.1 | 0.1 | 46.0 | 3565.9 | 5.7 | 17.8 | 565.7 | 20443.4 |
| ***L. alticolor*** | 46.9 | 3.3 | -18.0 | 4.4 | 17.6 | 25.1 | 362.2 | 107.9 | 1.2 | 106.7 | 4207.4 | 7.8 | 13.6 | 250.6 | 18102.2 |
| ***L. andinus*** | 65.0 | 7.9 | -24.1 | 4.4 | 16.4 | 24.7 | 64.1 | 22.2 | 0.7 | 21.5 | 4344.4 | 8.6 | 14.9 | 106.3 | 20832.1 |
| ***L. araucaniensis*** | 60.9 | 3.5 | -39.6 | 8.2 | 12.7 | 23.1 | 1309.0 | 232.0 | 32.8 | 199.2 | 1030.0 | 9.1 | 13.6 | 538.3 | 14297.8 |
| ***L. atacamensis*** | 65.7 | 3.0 | -26.9 | 13.5 | 11.2 | 17.9 | 23.6 | 7.6 | 0.1 | 7.5 | 1383.9 | 10.1 | 14.7 | 19.6 | 20565.4 |
| *L. barbarae* | 55.0 | 3.9 | -22.7 | 12.5 | 15.8 | 25.4 | 201.0 | 44.0 | 1.0 | 43.0 | 2520.7 | 3.8 | 18.7 | 243.4 | 19622.5 |
| ***L. bellii*** | 73.8 | 2.0 | -33.4 | 6.1 | 14.1 | 24.7 | 519.1 | 115.6 | 4.3 | 111.2 | 2432.4 | 9.2 | 43.3 | 260.9 | 17129.7 |
| ***L. bibronii*** | 56.0 | 4.0 | -42.7 | 9.4 | 12.7 | 25.7 | 305.2 | 48.3 | 12.1 | 36.2 | 759.7 | 6.2 | 10.0 | 342.1 | 14265.7 |
| ***L. boulengeri*** | 63.4 | 4.8 | -43.5 | 9.4 | 12.5 | 25.6 | 252.6 | 38.1 | 10.5 | 27.6 | 628.2 | 5.4 | 5.8 | 325.4 | 13949.9 |
| ***L. buergeri*** | 93.2 | 3.0 | -36.0 | 7.2 | 15.2 | 27.7 | 591.8 | 110.5 | 14.9 | 95.7 | 2129.1 | 11.3 | 19.0 | 348.9 | 16082.3 |
| ***L. ceii*** | 83.6 | 4.0 | -40.1 | 8.4 | 13.9 | 26.0 | 483.0 | 89.4 | 12.1 | 77.4 | 1179.5 | 10.0 | 9.0 | 425.7 | 14730.5 |
| ***L. chiliensis*** | 90.2 | 2.5 | -36.2 | 10.7 | 12.4 | 22.4 | 1007.4 | 202.5 | 16.0 | 186.5 | 526.4 | 13.3 | 40.0 | 531.6 | 15265.0 |
| *L. chillanensis* | 69.4 | 4.0 | -37.5 | 6.4 | 14.3 | 25.2 | 1050.3 | 202.8 | 19.3 | 183.5 | 1758.8 | 8.6 | 22.0 | 427.2 | 14718.9 |
| ***L. coeruleus*** | 62.1 | 0.0 | -38.2 | 6.7 | 14.6 | 25.6 | 875.0 | 163.0 | 17.5 | 145.5 | 1790.3 | 13.2 | 13.5 | 399.1 | 13919.8 |
| *L. constanzae* | 64.4 | 3.5 | -23.0 | 11.7 | 15.7 | 24.2 | 39.8 | 20.9 | 0.0 | 20.9 | 2732.8 | 5.2 | 11.1 | 50.6 | 21604.5 |
| *L. cristiani* | 73.7 | 0.0 | -35.6 | 8.8 | 14.5 | 26.8 | 1009.0 | 211.0 | 16.0 | 195.0 | 1573.0 | 6.7 | 26.7 | 393.7 | 15271.8 |
| *L. curicensis* | 58.9 | 2.4 | -34.4 | 5.3 | 14.4 | 25.6 | 771.7 | 166.8 | 8.7 | 158.2 | 2520.7 | 9.3 | 34.6 | 320.6 | 16527.0 |
| ***L. curis*** | 85.8 | 1.7 | -35.0 | 3.8 | 14.5 | 25.9 | 744.0 | 151.0 | 12.3 | 138.8 | 2649.8 | 7.4 | 32.9 | 312.5 | 16444.9 |
| ***L. cyanogaster*** | 62.4 | 2.4 | -39.0 | 10.5 | 10.7 | 19.8 | 1748.7 | 304.8 | 42.3 | 262.5 | 351.9 | 13.2 | 9.1 | 563.1 | 13832.3 |
| ***L. darwinii*** | 58.6 | 6.2 | -37.5 | 14.5 | 14.6 | 29.8 | 254.3 | 35.8 | 10.2 | 25.6 | 507.1 | 7.4 | 4.5 | 321.0 | 16467.2 |
| ***L. eleodori*** | 59.4 | 5.0 | -29.3 | 4.7 | 13.8 | 22.1 | 112.5 | 17.5 | 4.2 | 13.3 | 3444.3 | 15.4 | 22.4 | 54.4 | 20915.4 |
| ***L. elongatus*** | 82.0 | 4.0 | -40.7 | 8.3 | 13.6 | 26.5 | 376.9 | 69.0 | 10.8 | 58.2 | 1133.0 | 8.2 | 8.3 | 421.7 | 14842.7 |
| *L. erguetae* | 64.2 | 5.5 | -22.3 | 3.0 | 18.0 | 26.9 | 54.0 | 25.3 | 0.0 | 25.3 | 4632.0 | 6.3 | 15.6 | 60.8 | 20155.0 |
| ***L. escarchadosi*** | 82.3 | 6.6 | -50.4 | 6.1 | 10.2 | 21.5 | 210.7 | 24.8 | 12.0 | 12.8 | 385.8 | 6.3 | 6.8 | 390.7 | 10626.8 |
| ***L. fabiani*** | 79.3 | 5.6 | -23.2 | 13.4 | 15.3 | 23.9 | 43.3 | 21.8 | 0.0 | 21.8 | 2346.5 | 5.4 | 5.4 | 59.1 | 21726.6 |
| *L. filiorum* | 94.9 | 5.5 | -21.8 | 8.5 | 16.2 | 24.4 | 33.0 | 18.4 | 0.0 | 18.4 | 3264.3 | 4.9 | 10.6 | 7.2 | 21391.5 |
| *L. fitzgeraldi* | 55.4 | 2.4 | -32.7 | 2.5 | 13.7 | 24.5 | 325.7 | 72.2 | 6.5 | 65.7 | 3120.8 | 12.3 | 30.8 | 133.3 | 18209.1 |
| *L. flavipiceus* | 85.3 | 0.0 | -36.0 | 4.6 | 14.9 | 26.7 | 642.0 | 121.3 | 15.0 | 106.3 | 2494.0 | 8.6 | 15.7 | 344.8 | 15948.4 |
| *L. foxi* | 81.1 | 5.1 | -22.7 | 9.2 | 16.3 | 24.7 | 32.6 | 18.9 | 0.0 | 18.9 | 3316.0 | 5.5 | 13.9 | 24.3 | 21506.5 |
| ***L. fuscus*** | 45.9 | 2.6 | -33.1 | 11.3 | 14.3 | 24.5 | 454.9 | 107.9 | 2.2 | 105.7 | 1422.9 | 14.2 | 28.9 | 314.0 | 17489.3 |
| ***L. gravenhorstii*** | 56.6 | 2.5 | -33.6 | 15.1 | 14.2 | 26.9 | 430.8 | 109.4 | 1.4 | 108.0 | 462.8 | 14.0 | 4.9 | 438.4 | 16588.7 |
| *L. hajeki* | 64.5 | 6.4 | -21.9 | 7.0 | 16.8 | 25.3 | 41.1 | 21.3 | 0.0 | 21.3 | 3619.7 | 5.1 | 16.2 | 20.0 | 21074.7 |
| ***L. hatcheri*** | 64.3 | 0.0 | -48.5 | 5.9 | 10.0 | 21.8 | 239.3 | 31.0 | 13.0 | 18.0 | 770.6 | 7.7 | 11.2 | 399.0 | 11179.5 |
| ***L. hermannunezi*** | 54.1 | 8.0 | -37.5 | 6.6 | 14.6 | 25.6 | 1048.8 | 198.8 | 20.2 | 178.6 | 1819.4 | 10.5 | 12.9 | 371.7 | 15267.7 |
| ***L. irregularis*** | 78.9 | 8.9 | -24.0 | 7.4 | 17.4 | 27.6 | 134.4 | 40.6 | 0.0 | 40.6 | 3797.5 | 6.4 | 14.0 | 548.8 | 20330.1 |
| ***L. isabelae*** | 75.4 | 2.4 | -26.4 | 8.2 | 11.3 | 16.4 | 30.6 | 6.2 | 0.4 | 5.8 | 3430.3 | 7.8 | 12.7 | 1.7 | 20985.7 |
| *L. jamesi* | 90.3 | 3.9 | -18.8 | 2.8 | 18.1 | 25.1 | 240.8 | 85.4 | 0.0 | 85.4 | 4307.2 | 4.2 | 15.6 | 90.7 | 18878.3 |
| ***L. josei*** | 63.6 | 3.9 | -35.8 | 8.6 | 15.7 | 29.4 | 339.0 | 49.0 | 17.0 | 32.0 | 1799.0 | 13.3 | 14.9 | 319.1 | 16364.9 |
| ***L. kingi*** | 86.5 | 7.6 | -47.7 | 9.0 | 10.8 | 23.2 | 224.6 | 28.5 | 12.1 | 16.5 | 471.0 | 4.5 | 4.5 | 262.0 | 11947.9 |
| ***L. kolengh*** | 61.9 | 0.0 | -46.9 | 3.2 | 9.6 | 20.6 | 364.7 | 43.0 | 14.4 | 28.6 | 1060.3 | 9.6 | 19.5 | 455.5 | 11469.0 |
| ***L. koslowskyi*** | 61.6 | 5.7 | -28.1 | 17.1 | 16.0 | 31.2 | 227.4 | 54.3 | 2.8 | 51.5 | 1325.1 | 30.8 | 12.2 | 374.1 | 18452.4 |
| ***L. kriegi*** | 98.4 | 3.5 | -40.2 | 8.2 | 14.0 | 26.6 | 478.2 | 86.4 | 12.2 | 74.1 | 1208.2 | 8.7 | 10.3 | 410.1 | 14867.8 |
| ***L. laurenti*** | 51.8 | 7.1 | -29.0 | 17.1 | 16.5 | 32.5 | 210.6 | 51.5 | 2.0 | 49.5 | 1243.9 | 26.7 | 6.4 | 204.1 | 18018.3 |
| ***L. lemniscatus*** | 48.8 | 2.4 | -35.1 | 12.2 | 12.9 | 23.4 | 852.6 | 181.0 | 10.8 | 170.2 | 633.1 | 12.9 | 14.0 | 422.6 | 16050.9 |
| ***L. leopardinus*** | 88.0 | 1.7 | -33.3 | 5.7 | 14.0 | 24.6 | 495.0 | 110.8 | 4.2 | 106.6 | 2207.8 | 8.9 | 44.6 | 250.5 | 17119.9 |
| ***L. lineomaculatus*** | 58.3 | 0.0 | -47.5 | 7.4 | 10.6 | 22.4 | 299.7 | 43.0 | 12.8 | 30.2 | 617.0 | 6.7 | 7.2 | 351.0 | 11789.3 |
| *L. lorenzmuelleri* | 87.8 | 3.0 | -29.9 | 0.4 | 13.4 | 20.9 | 140.2 | 25.0 | 5.4 | 19.6 | 3757.2 | 7.4 | 30.9 | 35.5 | 20141.1 |
| ***L. lutzae*** | 76.8 | 5.8 | -22.9 | 22.9 | 8.0 | 14.0 | 1179.4 | 155.8 | 44.6 | 111.2 | 83.4 | 5.9 | 4.7 | 804.8 | 14743.6 |
| *L. maldonadae* | 84.3 | 2.5 | -30.6 | 4.0 | 13.3 | 21.4 | 147.3 | 32.4 | 2.6 | 29.9 | 3082.0 | 6.6 | 30.7 | 120.4 | 19569.2 |
| *L. melaniceps* | 90.4 | 3.0 | -29.4 | 15.2 | 9.0 | 15.7 | 71.0 | 19.0 | 0.0 | 19.0 | 1.0 | 5.6 | 16.7 | 90.6 | 15125.1 |
| ***L. melanops*** | 81.5 | 8.5 | -41.6 | 11.9 | 13.7 | 28.2 | 193.3 | 21.8 | 11.9 | 10.0 | 538.3 | 2.7 | 3.1 | 217.5 | 15153.6 |
| ***L. monticola*** | 62.2 | 2.3 | -33.5 | 9.3 | 14.6 | 25.3 | 549.3 | 125.0 | 3.8 | 121.1 | 1813.0 | 11.5 | 34.8 | 294.0 | 17471.6 |
| *L. moradoensis* | 59.1 | 2.0 | -33.8 | 2.3 | 14.2 | 25.1 | 543.8 | 108.4 | 9.0 | 99.4 | 2920.2 | 7.4 | 35.6 | 175.7 | 16510.4 |
| ***L. morenoi*** | 82.3 | 9.0 | -39.8 | 10.0 | 14.0 | 26.7 | 337.7 | 66.3 | 10.1 | 56.2 | 867.0 | 10.3 | 6.8 | 435.4 | 15555.6 |
| ***L. multimaculatus*** | 60.6 | 8.1 | -38.6 | 14.2 | 10.6 | 23.6 | 719.9 | 80.5 | 42.5 | 38.0 | 28.0 | 5.9 | 0.8 | 630.7 | 15564.4 |
| ***L. neuquensis*** | 57.8 | 0.0 | -38.3 | 7.8 | 14.8 | 26.2 | 718.0 | 135.5 | 14.5 | 121.0 | 1631.3 | 14.2 | 16.6 | 386.1 | 14948.3 |
| *L. nigriceps* | 90.3 | 5.9 | -24.8 | 6.9 | 14.8 | 21.1 | 23.0 | 7.5 | 0.0 | 7.5 | 3835.0 | 6.6 | 14.1 | 37.0 | 21082.2 |
| ***L. nigromaculatus*** | 64.7 | 2.5 | -27.5 | 15.9 | 10.4 | 17.9 | 36.3 | 12.1 | 0.0 | 12.1 | 407.1 | 6.0 | 10.5 | 34.8 | 17524.0 |
| ***L. nigroviridis*** | 70.4 | 3.4 | -33.4 | 8.3 | 14.4 | 24.8 | 519.7 | 119.0 | 3.8 | 115.3 | 2114.1 | 13.6 | 35.4 | 287.8 | 17337.4 |
| ***L. nitidus*** | 91.5 | 2.1 | -32.5 | 10.9 | 12.8 | 22.6 | 398.6 | 94.9 | 2.5 | 92.4 | 1380.4 | 11.4 | 25.0 | 257.4 | 17241.3 |
| ***L. olongasta*** | 66.0 | 6.7 | -30.3 | 15.4 | 16.2 | 31.9 | 132.8 | 30.6 | 2.2 | 28.3 | 1221.4 | 30.2 | 6.5 | 121.6 | 18873.7 |
| ***L. ornatus*** | 68.4 | 7.7 | -22.5 | 8.3 | 19.0 | 30.2 | 197.7 | 56.8 | 0.1 | 56.8 | 3793.9 | 7.3 | 12.8 | 543.7 | 19468.8 |
| *L. pantherinus* | 64.3 | 5.4 | -20.5 | 4.8 | 17.6 | 25.7 | 110.6 | 46.3 | 0.0 | 46.3 | 4026.1 | 6.1 | 17.5 | 44.1 | 20197.4 |
| *L. patriciaiturrae* | 90.8 | 5.6 | -26.5 | 7.2 | 11.6 | 16.8 | 35.8 | 6.6 | 0.8 | 5.8 | 3604.0 | 7.8 | 15.0 | 1.6 | 21107.9 |
| ***L. paulinae*** | 53.0 | 4.3 | -22.5 | 12.6 | 14.6 | 22.6 | 26.6 | 16.3 | 0.0 | 16.3 | 2338.1 | 4.8 | 6.5 | 71.1 | 21863.9 |
| ***L. petrophilus*** | 92.0 | 3.4 | -41.5 | 10.0 | 14.1 | 28.5 | 201.9 | 26.5 | 10.5 | 16.0 | 897.7 | 3.3 | 4.8 | 256.8 | 15012.3 |
| ***L. pictus*** | 64.7 | 2.9 | -40.6 | 8.7 | 10.2 | 19.4 | 1714.3 | 272.4 | 54.7 | 217.7 | 703.7 | 9.8 | 14.8 | 589.8 | 13029.5 |
| ***L. platei*** | 53.8 | 3.0 | -28.5 | 14.3 | 10.7 | 18.3 | 69.9 | 19.7 | 0.2 | 19.5 | 707.1 | 7.2 | 15.8 | 76.3 | 17423.8 |
| *L. pleopholis* | 72.7 | 5.5 | -18.2 | 1.9 | 18.5 | 25.4 | 312.8 | 106.4 | 0.0 | 106.4 | 4517.9 | 4.5 | 9.3 | 126.4 | 18228.3 |
| ***L. pseudolemniscatus*** | 47.5 | 2.4 | -31.0 | 13.7 | 11.1 | 19.4 | 175.7 | 45.2 | 0.1 | 45.2 | 673.9 | 10.5 | 14.8 | 194.9 | 17821.4 |
| *L. puritamensis* | 94.9 | 6.5 | -22.8 | 4.5 | 17.3 | 25.7 | 50.8 | 23.3 | 0.0 | 23.3 | 4161.5 | 4.6 | 23.2 | 66.9 | 20831.4 |
| ***L. quilmes*** | 59.1 | 5.7 | -25.8 | 13.5 | 14.0 | 25.2 | 187.6 | 49.5 | 0.2 | 49.3 | 2248.8 | 9.2 | 21.8 | 706.2 | 18809.1 |
| *L. ramonensis* | 89.9 | 3.4 | -33.5 | 9.3 | 14.7 | 25.4 | 574.0 | 131.3 | 3.7 | 127.7 | 2277.7 | 10.7 | 36.1 | 309.7 | 17140.5 |
| ***L. riojanus*** | 55.3 | 8.4 | -30.8 | 17.0 | 15.5 | 32.2 | 198.4 | 45.1 | 2.3 | 42.9 | 1011.0 | 12.8 | 6.5 | 154.0 | 17680.6 |
| *L. robertoi* | 62.7 | 5.5 | -29.8 | 0.3 | 13.4 | 20.7 | 136.8 | 23.7 | 5.5 | 18.2 | 3873.8 | 8.1 | 31.2 | 37.9 | 20340.3 |
| *L. rosenmanni* | 70.7 | 7.0 | -27.2 | 3.2 | 12.6 | 18.5 | 71.7 | 11.6 | 2.2 | 9.4 | 4221.8 | 7.2 | 15.4 | 3.9 | 20784.6 |
| ***L. rothi*** | 93.3 | 9.2 | -41.2 | 8.1 | 13.3 | 26.4 | 395.0 | 67.3 | 12.4 | 54.9 | 1096.7 | 6.3 | 7.2 | 362.9 | 14653.6 |
| ***L. ruibali*** | 57.5 | 5.1 | -31.9 | 8.3 | 14.5 | 26.3 | 176.0 | 24.5 | 7.9 | 16.5 | 2371.2 | 19.3 | 19.3 | 195.7 | 20107.0 |
| ***L. sagei*** | 75.1 | 8.3 | -39.7 | 9.6 | 14.0 | 26.5 | 326.9 | 65.1 | 9.0 | 56.1 | 994.9 | 11.3 | 6.4 | 441.6 | 15574.5 |
| ***L. salinicola*** | 61.8 | 7.9 | -27.7 | 16.3 | 16.2 | 29.7 | 157.0 | 39.1 | 2.0 | 37.1 | 1535.4 | 30.7 | 9.3 | 266.0 | 19107.8 |
| ***L. sarmientoi*** | 85.5 | 6.5 | -50.5 | 6.9 | 10.1 | 21.1 | 203.8 | 24.4 | 11.2 | 13.2 | 286.8 | 7.9 | 4.0 | 375.6 | 10632.2 |
| ***L. scapularis*** | 64.8 | 7.3 | -26.8 | 15.0 | 13.8 | 26.4 | 180.6 | 45.7 | 0.9 | 44.8 | 1997.6 | 23.3 | 20.9 | 700.8 | 19484.2 |
| ***L. schroederi*** | 59.3 | 2.3 | -35.0 | 9.9 | 13.0 | 22.9 | 898.6 | 191.0 | 11.9 | 179.1 | 1304.8 | 14.7 | 24.1 | 392.3 | 16293.3 |
| ***L. scolaroi*** | 63.1 | 8.3 | -46.8 | 3.4 | 9.4 | 20.2 | 413.3 | 47.9 | 16.6 | 31.2 | 936.2 | 9.2 | 19.8 | 451.4 | 11538.1 |
| ***L. silvai*** | 59.8 | 3.0 | -29.1 | 15.6 | 9.5 | 16.4 | 63.0 | 18.0 | 0.0 | 18.0 | 220.7 | 3.4 | 6.5 | 60.6 | 15136.3 |
| ***L. silvanae*** | 74.2 | 0.0 | -47.0 | 4.3 | 10.3 | 22.3 | 220.8 | 29.3 | 7.3 | 22.0 | 1248.9 | 10.0 | 7.0 | 285.0 | 11486.8 |
| ***L. stolzmanni*** | 55.2 | 6.5 | -20.2 | 16.4 | 9.9 | 16.7 | 0.0 | 0.0 | 0.0 | 0.0 | 919.0 | 12.2 | 8.3 | 0.0 | 21358.5 |
| ***L. tenuis*** | 57.0 | 2.6 | -36.2 | 11.5 | 12.8 | 23.3 | 1109.2 | 222.3 | 18.2 | 204.1 | 630.4 | 12.5 | 13.5 | 480.0 | 15480.1 |
| ***L. thermarum*** | 78.4 | 0.0 | -35.3 | 3.7 | 14.5 | 26.0 | 735.4 | 147.4 | 13.2 | 134.2 | 2614.6 | 7.0 | 23.4 | 339.4 | 16649.0 |
| ***L. tregenzai*** | 82.5 | 0.0 | -37.8 | 7.0 | 14.7 | 25.9 | 1021.0 | 191.0 | 20.0 | 171.0 | 1891.0 | 11.6 | 15.5 | 379.8 | 14380.3 |
| *L. valdesianus* | 88.9 | 1.7 | -33.9 | 1.4 | 14.2 | 25.2 | 539.8 | 105.0 | 10.0 | 95.0 | 3144.0 | 7.1 | 33.2 | 172.4 | 16456.0 |
| ***L. vallecurensis*** | 61.6 | 6.0 | -29.7 | 1.0 | 13.6 | 21.4 | 142.0 | 21.7 | 6.3 | 15.3 | 3826.0 | 17.9 | 24.6 | 90.9 | 20318.4 |
| ***L. velosoi*** | 50.0 | 2.5 | -26.7 | 15.7 | 11.3 | 18.7 | 18.7 | 6.2 | 0.0 | 6.2 | 811.0 | 8.2 | 9.3 | 21.0 | 21088.8 |
| ***L. wiegmannii*** | 56.1 | 6.1 | -34.1 | 16.2 | 13.0 | 27.2 | 708.8 | 93.4 | 29.4 | 63.9 | 382.0 | 6.7 | 4.0 | 640.3 | 16567.7 |
| ***L. zapallarensis*** | 87.7 | 3.5 | -30.9 | 14.9 | 9.9 | 17.5 | 177.5 | 49.6 | 0.0 | 49.6 | 291.5 | 10.4 | 12.7 | 200.7 | 15356.8 |
| ***L. zullyiae*** | 73.9 | 7.4 | -46.8 | 4.3 | 9.5 | 20.5 | 354.8 | 43.5 | 13.3 | 30.2 | 901.6 | 9.7 | 19.4 | 441.6 | 11987.2 |
